# Amino Acid Composition of Dried Bovine Dairy Powders from a Range of Product Streams

**DOI:** 10.3390/foods13233901

**Published:** 2024-12-03

**Authors:** Simon R. Gilmour, Stephen E. Holroyd, Maher D. Fuad, Dave Elgar, Aaron C. Fanning

**Affiliations:** Fonterra Research and Development Centre, Dairy Farm Road, Palmerston North 4442, New Zealand; steve.holroyd@fonterra.com (S.E.H.); maher.fuad@fonterra.com (M.D.F.); aaron.fanning@fonterra.com (A.C.F.)

**Keywords:** dairy protein, amino acid, whey protein concentrate, milk powder variability, protein fractionation, nutritional composition

## Abstract

The amino acid (AA) content of multiple samples of various dairy powders was determined, providing a comprehensive evaluation of the differences in AA profiles attributable to distinct manufacturing processes. Products examined included whole milk powder (WMP), skim milk powder (SMP), cheese whey protein concentrate (WPC-C), lactic acid casein whey protein concentrate (WPC-L), high-fat whey protein concentrate (WPC-HF), hydrolyzed whey protein concentrate (WPH), whey protein isolate (WPI), and demineralized whey protein (D90). WMP and SMP exhibited broadly similar AA profiles, with minor differences likely due to the minimal milk fat protein content, which is nearly absent from SMP. Comparative analysis of WPC-C and WPC-L indicated higher levels of threonine, serine, glutamic acid, and proline in WPC-C but lower levels of tyrosine, phenylalanine, and tryptophan, attributed to the different methods of separation from casein proteins. WPI and WPC-HF originate from similar sweet whey streams but follow divergent processing methods; consequent on this were variations in the levels of all AAs except histidine. The nanofiltration step in D90 production retains its non-protein nitrogen content and affects its AA profile; consequently, D90 consistently exhibited lower AA levels than WPC-C. These findings underscore the significant impact of manufacturing processes on dairy powder AA composition.

## 1. Introduction

Dairy powders serve as nutrient-dense sources of high-quality nutrition and are integral ingredients in a wide array of foods consumed by individuals across all age groups. As global awareness of the critical role of nutrition in maintaining health and wellness continues to rise [1], the demand for dairy products is also increasing, a trend anticipated to persist over the next decade [2]. Dairy proteins are recognized for their high quality, attributed to their superior digestibility and essential amino acid (AA) content [3].

However, it is crucial to acknowledge that not all dairy powders are interchangeable as sources of high-quality protein. Each type of powder is produced through distinct manufacturing processes, resulting in variability in their comparative AA content. This variability determines the suitability of each powder for different applications, such as a branched-chain amino acid (leucine, isoleucine, and valine)-rich protein source for sports nutrition products [4,5], a low-mineral protein source for infant formulas to prevent excessive renal solute load [6], or a highly concentrated protein source with low lactose content for consumers with lactose intolerance.

This paper explores two primary factors that impact the AA content of dairy powders: the fractionation of different protein species within dairy and the level of non-protein nitrogen (NPN) present in the powder. The amino acid data presented are normalized to mg AA/g crude protein (Total nitrogen (TN) × 6.38).

Dairy proteins are typically categorized into two main types based on relative solubility: casein proteins (contained in colloidal micelles) that constitute approximately 80% of total protein, and whey proteins that make up the remaining 20%. However, there is greater diversity within dairy proteins than this high-level categorization suggests. The principal casein proteins include α_S1_-casein, α_S2_-casein, β-casein, and κ-casein, while the principal whey proteins are α-lactalbumin (α-Lac), β-lactoglobulin (β-Lg), bovine serum albumin (BSA), and immunoglobulins [7]. Additionally, there are numerous minor proteins, such as those associated with the milk fat globular membrane (MFGM), which is a phospholipid tri-layer encapsulating milk fat [8].

Each of these proteins has a unique AA profile that contributes to the overall AA profile of total milk protein. The separation of these proteins through various manufacturing processes alters the resultant AA profile. For instance, whole milk powder (WMP) and skim milk powder (SMP) contain both whey and casein proteins, while products derived from whey protein concentrate (WPC) contain no intact casein proteins but still contain large casein peptide fragments. Whey protein concentrates also vary in their levels of caseinomacropeptides (CMP); products from rennet casein whey and cheese whey have high levels of CMP, whereas those from milk acidification have low levels of CMP [9,10]. Furthermore, high-fat products like high-fat whey protein concentrate (WPC-HF) contain higher levels of MFGM-associated proteins, while low-fat products like whey protein isolate (WPI) contain little due to their low fat content.

Another significant factor influencing the variability of AA profiles in dairy powders is the NPN content. Higher or lower NPN levels than typically found in dairy products can skew the measured protein level and AA content when it is expressed on a mg/g crude protein basis. This discrepancy arises from the commonly used Kjeldahl method, which measures total nitrogen and applies a conversion factor to estimate total protein. For dairy products, a total nitrogen conversion factor of 6.38 is used, while other foods like soy protein use 6.25 [11]. These assumptions may become less accurate when milk protein is fractionated into different product streams containing more nitrogen from sources such as urea or nitrogen-containing vitamins (e.g., B12 or B2). This discrepancy can be addressed by measuring the NPN in a sample and adjusting the total protein calculation accordingly. However, this practice is not standard in commercial settings as it is not required for labelling purposes. This paper seeks to explain the real and apparent differences in AA profiles of commercially available dairy powders without assessing NPN values.

Given that each AA plays a vital and often unique role in human health and wellbeing, understanding this variation is valuable for product design and formulation. Accurate knowledge of the AA content of protein ingredients used in infant formula production is essential, as AA requirements in such products are clearly defined by regulations [12]. Historically, manufacturers could simply add more protein to meet AA requirements, but there is now a movement towards lower protein formulations that more closely match the protein content of human breastmilk [13], necessitating greater consideration of AA content per gram of protein.

Recent years have seen considerable interest in determining the AA composition and protein quality of both animal and non-animal sources [14,15,16,17]. This paper aims to support these efforts by contributing to the publicly available knowledge of AA availability in the food supply. It outlines the AA content of multiple dairy powders, including whole milk powder (WMP), skim milk powder (SMP), cheese whey protein concentrate (WPC-C), lactic acid casein whey protein concentrate (WPC-L), high-fat whey protein concentrate (WPC-HF), hydrolyzed whey protein concentrate (WPH), whey protein isolate (WPI), and demineralized whey protein (D90), while providing insights into the reasons for the variations observed among these different dairy products.

## 2. Materials and Methods

### 2.1. Dairy Powder Samples Selection

Samples of various dairy powders were collected from retention samples of commercially produced batches. A comprehensive list of available retention samples was generated, and batches were categorized into three groups, each spanning four consecutive months of the year. Efforts were made to obtain samples from fifteen separate batches for each powder type; however, only four batches were available for whole milk powder (WMP), eleven for demineralized whey protein (D90), and one for hydrolyzed whey protein concentrate (WPH).

Batches were then randomly selected from each four-month grouping, and samples were collected to obtain a representative sample that accounted for any seasonal variation. Unfortunately, this was not possible for WMP and WPH, as the available WMP batches were all manufactured in the same month (November 2018), and only one batch of WPH was available (February 2017). Given the limited availability of WPH, fifteen samples were taken from the single batch to provide an indication of the variability of the AA test.

All samples were produced from New Zealand-sourced milk, with the exception of the demineralized whey protein (D90) samples, which were of European origin.

### 2.2. Total Protein Analysis

Internal data from the time of manufacture were utilized for this study. Protein content was measured using near-infrared spectroscopy (NIR) [18] during production runs at the factories producing the powders. The NIR instruments were calibrated daily by comparison with results from Kjeldahl testing [19,20]. The accuracy of both methods was ensured by testing reference products with known values and duplicating at least one sample for each product type.

### 2.3. Amino Acid Analysis

Amino acid (AA) analysis of protein products involved a two-step process: hydrolysis of proteins into AAs followed by the separation and measurement of the AAs using high-performance liquid chromatography (HPLC).

In brief, a 50 mg sample and internal standard (nor-leucine, Sigma N1398 or taurine, Sigma 30170 (Merck NZ, Auckland New Zealand)) were weighed and hydrolyzed in 6M HCl for 22 h at 110 °C. Prior to hydrolysis, the sulfur-containing AAs cystine and methionine were separately oxidized overnight to cysteic acid and methionine sulfone, respectively, and sodium metabisulfite hydrobromic acid was added to decompose the performic acid. During normal acid hydrolysis, glutamine and asparagine residues are converted to glutamic acid and aspartic acid, respectively, so the acids presented here include all residues acid [21]. Since tryptophan is destroyed by HCl acid hydrolysis, a separate analysis using a 150 mg sample for 20 h at 110 °C in 4.2 M NaOH was employed [22].

Amino acid analysis was performed using Ion Exchange Chromatography via Shimadzu 20Ai HPLC, with Pickering Pinnacle PCX ninhydrin post-column derivatization and UV/Vis detection (570, 440 nm). Ninhydrin derivatization solution was prepared fresh, held from light and under nitrogen. Analytical performance was monitored by a quality control standard. All samples were tested by calibration standards and corrected for Internal Standard recovery.

### 2.4. Statistical Analyses

All data entry and analysis were conducted using IBM SPSS Statistics (version 25). A *p*-value of less than 0.05 was considered significant. Data were checked for outliers and errors in entry and cleaned accordingly.

Descriptive statistics were performed for each variable. The normality of the distribution of the numerical variables was assessed. Means and standard deviations were used for normally distributed data. Frequencies and percentages were calculated for categorical variables.

Bivariable analyses were performed using independent *t*-tests to compare amino acids (mg/g protein or percentages of amino acids versus total; data are provided in Supplementary Materials Tables S1–S5) between different dairy powders. Assumptions of random sample independence, normality, and equality of variance were verified.

## 3. Results

Our findings clearly demonstrate a low variability in amino acid (AA) profiles for each product type (Table 1). This observation aligns with the expectation that the expression of proteins in bovine milk is a tightly regulated biological process, inherently minimizing variability [23]. Given the consistency of the AA profile in bovine milk, any observed variability within product types can likely be attributed either to the variability of the test methods employed to determine the AA profiles or to the manufacturing process itself.

**Figure 1:**
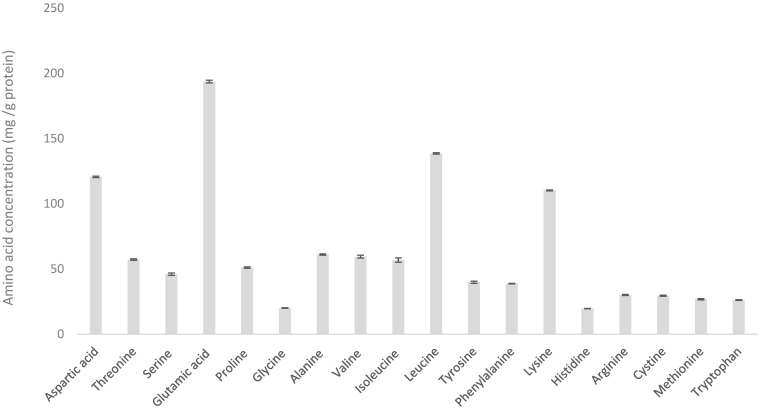
Amino acid profile of hydrolyzed whey protein concentrate (WPH).

To infer the variability introduced by the testing methods, we analyzed the hydrolyzed whey protein concentrate (WPH) results presented in this study (Figure 1). All 15 samples of WPH were derived from the same batch of powder and were tested in the same laboratory, providing a controlled basis for assessment. The analysis indicated that the testing variability was minimal, with the largest variations, as indicated by standard deviation (SD), observed in isoleucine (SD = 1.68), valine (SD = 1.16), glutamic acid (SD = 1.09), serine (SD = 0.94), and tyrosine (SD = 0.82). All other amino acids exhibited a standard deviation of SD ≤ 0.6 (Table 1).

Based on these findings, it is reasonable to infer that any significant variances exceeding those observed in the WPH samples are likely attributable to variability within the manufacturing process. With the exception of the WPH and whole milk powder (WMP), the samples analyzed were manufactured over multiple years and across various months within those years. The low variability observed within these product types suggests that the manufacturing process has a limited impact on the AA profile variability of each product type. The results also show some statistically significant mean differences (Tables S1–S5) between product groups. This analysis reveals clear differences observed between product types; the following comparisons outline these differences and explain how these differences arise.

### 3.1. Variability of WPH

Hydrolyzed whey protein concentrate (WPH) is derived from a whey protein stream produced via the acidification of milk, followed by enzymatic hydrolysis to break down the proteins into smaller peptides [24].

As previously noted, the 15 WPH samples analyzed all originated from the same batch and were tested in the same laboratory. Assuming the homogeneity of the sample, the amino acid (AA) content of each sample should be consistent. As illustrated in Figure 1, the variability within these samples is very low, indicating a high level of accuracy in the testing process.

### 3.2. Comparison of WMP and SMP

**Figure 2:**
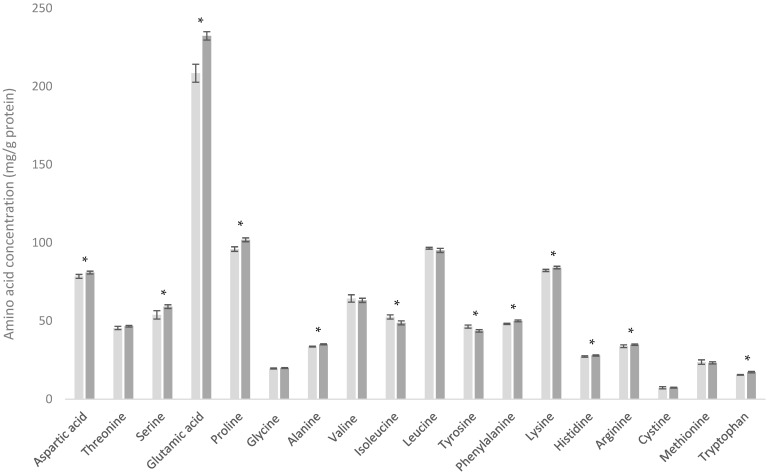
Amino acid profiles of whole milk powder (WMP) (■) and skim milk powder (SMP) (■). * Indicates statistical difference (*p* < 0.05).

On a per gram of protein basis, both whole milk powder (WMP) and skim milk powder (SMP) exhibited similar amino acid (AA) profiles (Figure 2). This is unsurprising as the primary distinction between the two products is that SMP undergoes processing to remove fat prior to drying, whereas WMP does not [24].

Our data, however, revealed differences between WMP and SMP. Analysis indicated that 12 of the 18 AAs were statistically different between the products (*p* ≤ 0.05). Nonetheless, the mean difference between them for each AA was minimal (less than 6 mg/g protein for 11 of the 12 AAs), suggesting that these differences are of little nutritional or functional significance.

There was, however, a notable variation in glutamic acid content (which represents both glutamate and glutamine). WMP had a mean glutamic acid value of 217 mg/g protein, while SMP had a mean value of 237 mg/g protein, with the mean difference between the samples being 23.93 mg/g protein. These observed differences may be attributed to the presence of milk fat globule membrane (MFGM) in WMP. MFGM is a phospholipid tri-layer that surrounds the fat globules in milk and is perforated with a variety of glycosylated and non-glycosylated proteins such as mucin 1 (MUC 1), xanthine oxidase (XO), CD36 (PAS 4), mucin 15 (PAS 3), butyrophilin (BTN), PAS 6/7, adipophilin (ADPH), and fatty acid binding protein (FABP) [8]. These proteins have been reported to make up between 22.3 and 28% of the total weight of MFGM [25,26]. The amino acid composition of these MFGM proteins show significant differences from other dairy proteins [27].

### 3.3. Comparison of WPC-C and WPC-L

The whey protein concentrates (WPC) analyzed in this study are produced using different methods which directly influence their resulting AA profiles. A key distinction lies in how the whey proteins are initially separated from casein proteins. Two common methods include (i) the addition of rennet or chymosin to milk (used in cheese making and rennet casein processes) and (ii) the action of acid on milk, either through direct acid addition or fermentation using lactic acid-producing bacteria [24]. Both methods result in the coagulation of casein proteins and their separation from whey proteins, albeit through different mechanisms.

The addition of rennet or chymosin enzymatically cleaves the κ-casein from the exterior of the casein micelle, causing aggregation of casein micelles. In contrast, the addition of acid neutralizes the negative charge of the κ-casein [28]. This disruption of the κ-casein peptide via these methods removes the steric repulsion provided by the negative charge on the κ-casein peptide [29], allowing the casein micelles to aggregate and form a curd.

When κ-casein is cleaved by rennet or chymosin, it produces insoluble para-κ-casein and water-soluble CMP [28]. The soluble CMP is associated with the whey protein fraction, while the para-κ-casein remains with the casein proteins. Conversely, when milk is acidified, the κ-casein is not cleaved into CMP and para-κ-casein; instead, it is retained in the aggregated casein micelle curd, not solubilizing into the whey protein stream [9].

**Figure 3:**
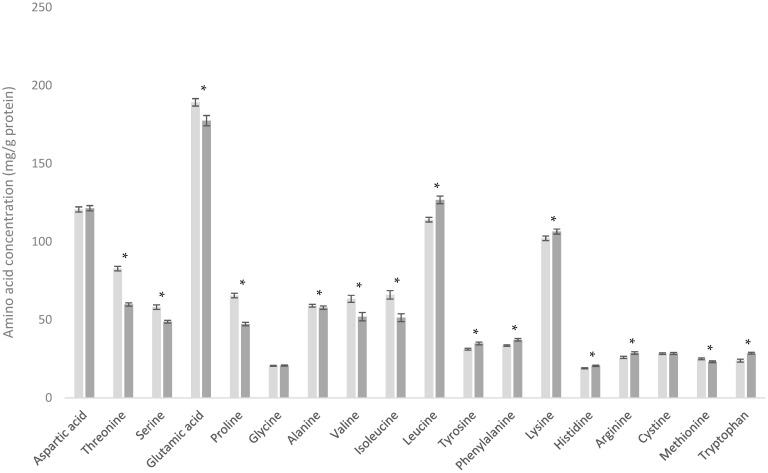
Amino acid profiles of cheese whey protein concentrate (WPC-C) (■) and lactic acid casein whey protein concentrate (WPC-L) (■). * Indicates statistical difference (*p* < 0.05).

CMP has an AA profile different from that of whey proteins, and is described as being rich in threonine, serine, glutamine (converted to glutamic acid during analysis), and proline while being depleted in tyrosine, phenylalanine, cystine, and tryptophan [30,31]. Therefore, the presence or absence of CMP in a whey protein stream impacts the overall AA content. This is evident when comparing WPC produced by renneting (WPC-C) and lactic acid casein whey protein concentrate (WPC-L) (Figure 3). The WPC-C analyzed here contains CMP due to the renneting process, while the WPC-L does not, as it is produced via acidification (although other casein peptides may arise during fermentation).

Threonine, serine, glutamic acid, and proline were present at higher levels in the CMP-containing WPC-C product compared with the WPC-L product, with mean differences of 22.92, 9.33, 11.73, and 18.27 mg/g protein, respectively. Tyrosine, phenylalanine, and tryptophan were lower in WPC-C, although the magnitude of these differences was smaller, with respective mean differences of −3.67, −3.71, and −4.74 mg/g protein, making these differences nutritionally irrelevant for most applications. Cystine showed no significant difference (*p* = 0.753) between the two products.

Interestingly, significant differences were also observed in the branched-chain amino acids (BCAAs) valine, isoleucine, and leucine, with mean differences of 11.45, 14.65, and −12.66 mg/g protein, respectively. This is notable as these BCAAs play key roles in metabolic processes such as glycogen synthesis [32,33] and muscle protein synthesis, with leucine being considered a key signaling molecule for muscle protein synthesis [31]. This makes BCAAs particularly important in products focused on muscle health, thereby being highly relevant to manufacturers of sports nutrition foods or supplements.

### 3.4. Comparison of WPI and WPC-HF

The production of whey protein isolate (WPI) from cheese whey typically employs either ion exchange (IX) or microfiltration (MF). Both processes yield a WPI stream devoid of fat and reduced in lactose, along with a co-product stream characterized by elevated fat content, commonly referred to as high-fat whey protein concentrate (WPC-HF).

**Figure 4:**
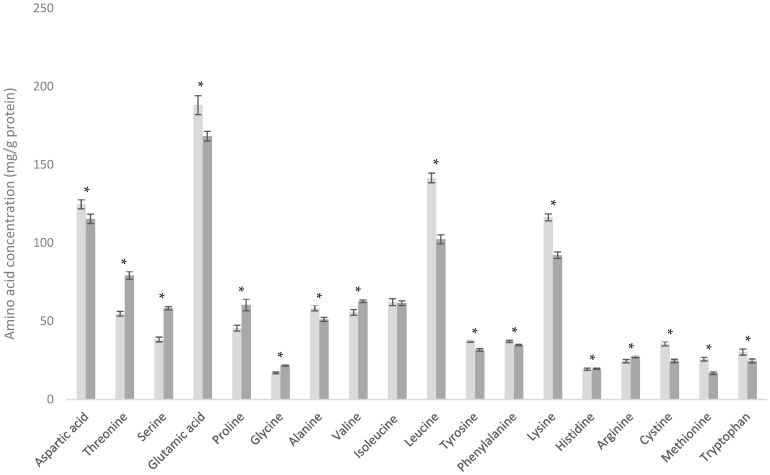
Amino acid profiles of whey protein isolate (WPI) (■) and high-fat whey protein concentrate (WPC-HF) (■). * Indicates statistical difference (*p* < 0.05).

The WPI and WPC-HF products analyzed in this study exhibited substantially different amino acid (AA) profiles (Figure 4). Although both originated from similar sweet whey (WPC-C) streams, the WPI was produced via an IX process, whereas the WPC-HF was produced via an MF process.

In the MF process, the permeate (the portion that passes through the membrane filter) becomes the WPI stream, while the retentate (the portion that does not pass through the membrane) becomes the WPC-HF stream [24]. Since the WPC-HF retentate retains all the fat from the original sweet whey, proteins present in the milk fat globule membrane (MFGM) are also retained in this stream. The WPC-HF analyzed here was produced via an MF process that included acidification and heating of the whey protein stream prior to the MF step, resulting in the precipitation of proteins such as α-Lac, BSA, and immunoglobulin G (IgG). Consequently, the WPC-HF becomes enriched in these components but depleted of proteins such as β-Lg and CMP. In the case of WPI produced via MF, the β-Lg and CMP would move into the accompanying WPI stream [34].

However, the WPI analyzed here was not produced from the microfiltration of a sweet whey stream but rather via an IX process. While the IX process also results in a WPI stream and a WPC-HF stream, the protein species are separated in such a way that the WPI, rather than the WPC-HF, is enriched in α-Lac and BSA [35]. The WPI retains an increased concentration of β-Lg, and the WPC-HF stream resulting from the IX process becomes enriched in CMP protein species. This results in the WPI having an AA profile somewhat similar to that of acid WPC, whereas a standard MF WPI process would typically yield a product more akin to cheese WPC. The IX process also separates the protein from the majority of NPN in the stream. The convention is to normalize amino acid data per g crude protein (TN × 6.38). Products that have a lower NPN content therefore have a higher true protein/crude protein ratio and will deliver more amino acids per gram of crude protein. Conversely, a protein powder with a higher NPN content will need to be formulated at a higher crude protein content to deliver the same amount of amino acids i.e., the NPN content effectively dilutes the mg AA/g crude protein.

These differences are clearly evidenced in Figure 4, where noticeable differences are seen between almost every AA, with the exception of histidine. The mean difference in histidine between the two products was −1.66 mg/g protein, with no significant difference observed (*p* = 0.113). If a comparison were made between a WPI and a WPC-HF both produced via the MF process, or both by the IX process, the AA profiles would be expected to be even more divergent, as only one of the two would be enriched in α-Lac and BSA rather than both, as is the case here.

### 3.5. Comparison of D90 and WPC-C

Demineralized whey (D90), as its name suggests, has a lower mineral content (e.g., sodium, chlorine, potassium, and magnesium) compared with regular whey proteins, making it a valuable ingredient for applications where mineral levels need to be carefully managed, such as in infant formulas. The demineralization process can be achieved via nanofiltration, electrolysis, ion exchange, or a combination of these methods [36]. The D90 product analyzed in this study was produced using a combination of nanofiltration and ion exchange processes and originates from a renneted sweet whey stream.

The protein-concentrating nanofiltration step results in the loss of some NPN, leading to a lower NPN content than that of the sweet whey stream from which it is derived. Consequently, D90 has a higher total AA content per gram of crude protein compared with a standard non-nano filtrated sweet whey powder. However, it still contains more NPN than a standard WPC80, as WPC80 undergoes ultrafiltration, which is more effective at removing NPN.

**Figure 5:**
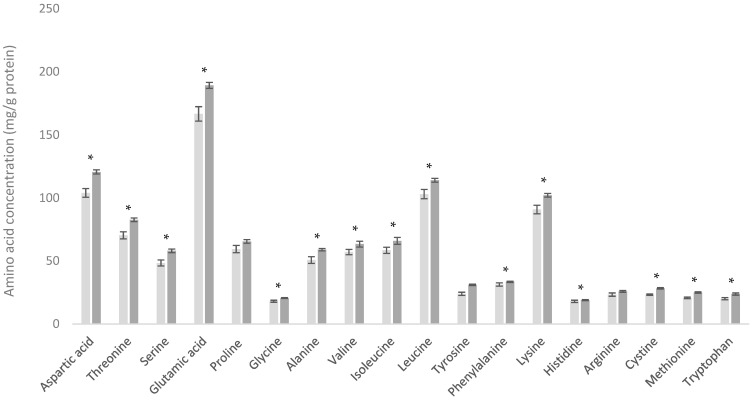
Amino acid profiles of demineralized whey (D90) (■) and cheese whey protein concentrate (WPC-C) (■). * Indicates statistical difference (*p* < 0.05).

**Figure 6:**
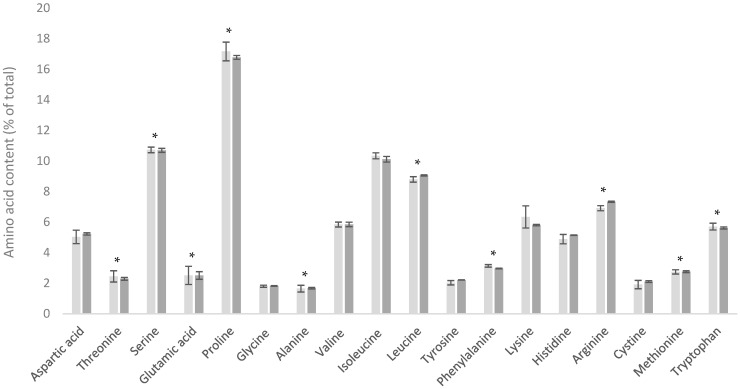
Amino acid profiles of demineralized whey (D90) (■) and cheese whey protein concentrate (WPC-C) (■) as a percentage of total amino acid content. * Indicates statistical difference (*p* < 0.05).

As shown in Figure 5, the AA content in the D90 product was consistently lower than in the WPC-C product. However, when comparing the AA contents of these products as a percentage of total AAs (which effectively corrects for NPN content), the resulting profiles were very similar (Figure 6). This similarity arises since in both the D90 and WPC80 processes, there is no significant loss or fractionation of the true protein components, but there are different retention levels for NPN, minerals, and lactose components.

## 4. Conclusions

The data presented in this paper clearly delineate the differences in AA profiles across a variety of dairy powders. These differences are relevant to the global trade in dairy protein-containing products where ingredient producers, regulators, and food processors create a range of valuable products. The observed differences can be attributed to two main factors: (i) changes in NPN distorting the apparent AA content when expressed on a per gram of protein basis, and (ii) the fractionation of different classes of proteins into distinct product streams.

When interpreting AA profiles, both of these factors should be considered. It is important to note that the distortion of AA profiles due to changes in NPN is merely a distortion. The absolute AA content remains unchanged; it only appears altered because protein testing is not a direct measurement of protein but rather a calculation based on total nitrogen content. Conversely, differences in AA profiles resulting from protein fractionation can lead to real and sometimes significant changes in AA profiles.

Protein categorization can be performed at multiple levels. For instance, milk proteins can be classified at the most basic level into whey proteins and casein proteins. These classifications can be further subdivided into four primary casein proteins (α_S1_-, α_S2_-, β-, and κ-caseins) and four primary whey proteins (α-Lac, β-Lg, BSA, and immunoglobulins). Each of these proteins comprises unique sequences of amino acids, meaning that when they are fractionated into different product streams, the resulting product will have an altered AA profile.

While tools such as protein quality scores (e.g., Digestible Indispensable Amino Acid Score (DIAAS)) provide useful insights into the overall quality of protein for consumers, understanding the specific differences in AA profiles of various powders is crucial when utilizing them as ingredients in nutritional applications. For example, manufacturers of food products for special medical purposes, infant formula manufacturers, or the regulatory authorities setting the nutrition requirements of these products require high quality nutritional data on a range of products.

This paper provides valuable insights into the nutritional differences between various dairy powders and offers context as to why these differences arise.

## Figures and Tables

**Table 1:** Amino acid content of dried dairy powders.

Amino Acid	Dairy Powder Amino Acid Content (mg/g Protein)							
	WMP	SMP	WPC-C	WPC-L	WPI	WPC-HF	D90	WPH
Alanine	33.6 ± 0.3	35.0 ± 0.4	58.9 ± 0.9	57.8 ± 1.1	58.3 ± 1.7	51.1 ± 1.25	50.7 ± 2.7	61.1 ± 0.5
Arginine	33.8 ± 0.9	34.8 ± 0.5	25.9 ± 0.7	28.7 ± 0.8	24.5 ± 1.0	27.4 ± 0.8	23.4 ± 1.3	30.1 ± 0.4
Aspartic acid	78.5 ± 1.3	80.9 ± 0.9	120.6 ± 1.7	121.4 ± 1.7	124.7 ± 2.9	115.4 ± 3.0	104.0 ± 3.4	120.7 ± 0.5
Cystine	7.3 ± 0.7	7.4 ± 0.3	28.3 ± 0.6	28.4 ± 0.7	35.5 ± 1.4	24.6 ± 1.0	23.4 ± 0.5	29.5 ± 0.4
Glutamic acid	208.5± 5.8	232.4 ± 2.7	189.2 ± 2.4	177.5 ± 3.3	188.1 ± 6.1	168.2 ± 3.1	166.6 ± 5.7	193.7 ± 1.1
Glycine	19.7 ± 0.4	20.0 ± 0.3	20.6 ± 0.3	20.7 ± 0.4	17.0 ± 0.6	21.8 ± 0.5	18.1 ± 0.8	20.2 ± 0.2
Histidine	27.2 ± 0.5	27.9 ± 0.4	19.0 ± 0.4	20.6 ± 0.4	19.3 ± 0.7	19.7 ± 0.4	18.0 ± 0.9	19.6 ± 0.1
Isoleucine	52.6 ± 1.3	48.8 ± 1.3	66.0 ± 2.7	51.3 ± 2.5	62.1 ± 2.2	61.5 ± 1.4	58.5 ± 2.5	56.9 ± 1.7
Leucine	96.5 ± 0.6	95.1 ± 1.3	114.1 ± 1.5	126.7 ± 2.4	141.5 ± 3.1	102.3 ± 2.9	103.0 ± 3.7	138.5 ± 0.6
Lysine	82.3 ± 0.7	84.2 ± 0.8	102.1 ± 1.5	106.4 ± 1.7	116.4 ± 2.2	92.1 ± 2.1	90.8 ± 3.4	110.3 ± 0.3
Methionine	23.8 ± 1.4	23.3 ± 0.7	25.0 ± 0.6	23.2 ± 0.5	25.8 ± 1.1	16.8 ± 0.8	20.7 ± 0.6	26.8 ± 0.4
Phenylalanine	48.1 ± 0.4	50.0 ± 0.6	33.5 ± 0.5	37.2 ± 0.9	37.1 ± 0.7	34.7 ± 0.5	31.4 ± 1.3	38.8 ± 0.2
Proline	96.0 ± 1.5	101.9 ± 1.2	65.5 ± 1.5	47.2 ± 1.1	45.5 ± 1.8	60.4 ± 3.7	59.5 ± 2.9	51.1 ± 0.6
Serine	53.9 ± 2.6	59.1 ± 1.2	58.1 ± 1.5	48.7 ± 0.9	38.3 ± 1.5	58.2 ± 1.0	48.4 ± 2.4	46.0 ± 0.9
Threonine	45.5 ± 1.0	46.6 ± 0.5	82.7 ± 1.5	59.8 ± 1.1	54.7 ± 1.5	79.2 ± 2.4	70.3 ± 2.8	57.3 ± 0.6
Tryptophan	15.6 ± 0.2	17.4 ± 0.5	23.8 ± 0.9	28.6 ± 0.6	30.2 ± 1.9	24.6 ± 1.1	20.1 ± 0.9	26.2 ± 0.3
Tyrosine	46.4 ± 1.0	43.7 ± 0.8	31.1 ± 0.6	34.8 ± 0.9	37.1 ± 0.7	31.6 ± 0.8	24.0 ± 1.3	39.9 ± 0.8
Valine	64.4 ± 2.4	63.2 ± 1.4	63.4 ± 2.3	51.9 ± 2.7	55.5 ± 1.8	62.7 ± 0.9	57.1 ± 2.1	59.5 ± 1.2
Total	1033.6	1071.8	1127.5	1070.6	1111.4	1052.3	988.0	1126.1

Abbreviations are as follows: WMP, whole milk powder; SMP, skim milk powder; WPC-C, cheese whey protein concentrate; WPC-L, lactic acid casein whey protein concentrate; WPI, whey protein isolate; WPC-HF, high-fat whey protein concentrate; D90, demineralized whey protein; WPH, hydrolyzed whey protein concentrate. Values (in mg/g protein, where protein = total nitrogen × 6.38) are means ± standard deviations (n = 15 for all powders except WMP and D90 where n = 4 and n = 11, respectively); values are rounded to one decimal place.

## Data Availability

The data presented in this study may only be available on request from the corresponding author due to potential intellectual property protection.

## Supplementary Materials

The following supporting information can be downloaded at: https://www.mdpi.com/article/10.3390/foods13233901/s1: Table S1. *t*-Test for whole milk powder (WMP) versus skim milk powder (SMP) (mg/g protein); Table S2. *t*-Test for cheese whey protein concentrate (WPC–C) versus lactic acid casein whey protein concentrate (WPC–L) (mg/g protein); Table S3. *t*-Test for whey protein isolate (WPI) versus hydrolyzed whey protein concentrate (Whey–HF) (mg/g protein); Table S4. *t*-Test for demineralized whey protein (D90) versus cheese whey protein concentrate (WPC–C) (mg/g protein); Table S5. *t*-Test for demineralized whey protein (D90) versus cheese whey protein concentrate (WPC–C) (AA %Total).
