# Supplementary material for: Amino Acid Composition of Dried Bovine Dairy Powders from a Range of Product Streams
Source: Foods. 2024 Dec 3;13(23):3901. doi: 10.3390/foods13233901 (PMC11640299; doi:10.3390/foods13233901)
Supplement: Supplementary file 1 [file foods-13-03901-s001.zip › foods-3300965-supplementary.pdf]

Supplementary Tables (t-Test)

Table S1. t-Test for whole milk powder (WMP) versus skim milk powder (SMP) (mg/g protein)

Amino acid	Product	M (sd)[1]	t (df)[2]	MD (95% C.I[3])	P value[4]
Aspartic Acid	WMP	78.51 (1.26)	-4.51 (17)	-2.42 (-3.56, -1.29)	0.000
	SMP	80.93 (0.88)			
Threonine	WMP	45.53 (1.04)	-2.06 (3.43)	-1.10 (-2.70, 0.49)	0.120
	SMP	46.63 (0.53)			
Serine	WMP	53.90 (2.65)	-3.83 (3.34)	-5.21 (-9.29, -1.12)	0.026
	SMP	59.11 (1.21)			
Glutamic Acid	WMP	208.48 (5.75)	-12.47 (17)	-23.93 (-27.98, -19.88)	0.000
	SMP	232.41 (2.65)			
Proline	WMP	95.97 (1.46)	-8.31 (17)	-5.91 (-7.41, -4.41)	0.000
	SMP	101.88 (1.22)			
Glycine	WMP	19.70 (0.39)	-1.59 (17)	-0.30 (-0.69, 0.10)	0.131
	SMP	20.00 (0.32)			
Alanine	WMP	33.58 (0.31)	-6.96 (17)	-1.46 (-1.91, -1.02)	0.000
	SMP	35.04 (0.39)			
Valine	WMP	64.38 (2.35)	1.37 (17)	1.22 (-0.65, 3.10)	0.187
	SMP	63.16 (1.36)			
Isoleucine	WMP	52.56 (1.33)	5.10 (17)	3.78 (2.22, 5.34)	0.000
	SMP	48.78 (1.31)			
Leucine	WMP	96.49 (0.60)	2.02 (17)	1.38 (-0.06, 2.82)	0.059
	SMP	95.11 (1.31)			
Tyrosine	WMP	46.37 (0.97)	5.63 (17)	2.68 (1.67, 3.68)	0.000
	SMP	43.69 (0.82)			
Phenylalanine	WMP	48.13 (0.43)	-6.18 (17)	-1.88 (-2.53, -1.24)	0.000
	SMP	50.02 (0.56)			
Lysine	WMP	82.32 (0.69)	-4.06 (17)	-1.84 (-2.80, -0.89)	0.001
	SMP	84.16 (0.83)			

Histidine	WMP	27.24 (0.50)	-2.78 (17)	-0.66 (-1.16, -0.16)	0.013
	SMP	27.90 (0.40)			
Arginine	WMP	33.80 (0.90)	-3.12 (17)	-1.03 (-1.73, -0.33)	0.006
	SMP	34.83 (0.49)			
Cystine	WMP	7.30 (0.70)	-0.35 (3.34)	-0.12 (-1.21, 0.96)	0.751
	SMP	7.43 (0.32)			
Methionine	WMP	23.78 (1.37)	0.65 (3.42)	0.46 (-1.65, 2.58)	0.555
	SMP	23.32 (0.69)			
Tryptophan	WMP	15.61 (0.19)	-6.98 (17)	-1.76 (-2.30, -1.23)	0.000
	SMP	17.37 (0.49)			

1. Mean (standard deviation)
2. t statistics (degrees of freedom)
3. Mean difference (95% confidence interval)
4. Independent t test

Table S2. t-Test for cheese whey protein concentrate (WPC–C) versus lactic acid casein whey protein concentrate (WPC–L) (mg/g protein)

Amino acid	Product	M (sd)[1]	t (df)[2]	MD (95% C.I[3])	P value[4]
Aspartic Acid	WPC	120.59 (1.66)	-1.28 (28)	-0.78 (-2.01, 0.46)	0.210
	WPC–L	121.36 (1.65)			
Threonine	WPC	82.68 (1.47)	49.07 (28)	22.92 (21.96, 23.88)	0.000
	WPC–L	59.76 (1.05)			
Serine	WPC	58.06 (1.45)	21.00 (23.65)	9.33 (8.41, 10.24)	0.000
	WPC–L	48.73 (0.92)			
Glutamic Acid	WPC	189.17 (2.38)	11.18 (28)	11.73 (9.58, 13.88)	0.000
	WPC–L	177.45 (3.29)			
Proline	WPC	65.51 (1.49)	38.28 (28)	18.27 (17.29, 19.24)	0.000
	WPC–L	47.24 (1.09)			
Glycine	WPC	20.59 (0.31)	-1.27 (28)	-0.16 (-0.42, 0.10)	0.216
	WPC–L	20.74 (0.37)			
Alanine	WPC	58.95 (0.90)	3.44 (28)	1.28 (0.52, 2.04)	0.002
	WPC–L	57.67 (1.12)			
Valine	WPC	63.39 (2.29)	12.52 (28)	11.45 (9.58, 13.32)	0.000
	WPC–L	51.94 (2.70)			
Isoleucine	WPC	65.97 (2.73)	15.39 (28)	14.65 (12.70, 16.60)	0.000
	WPC–L	51.32 (2.48)			
Leucine	WPC	114.05 (1.50)	-17.30 (28)	-12.66 (-14.16, -11.16)	0.000
	WPC–L	126.72 (2.40)			
Tyrosine	WPC	31.11 (0.56)	-13.10 (28)	-3.67 (-4.24, -3.09)	0.000
	WPC–L	34.77 (0.93)			
Phenylalanine	WPC	33.46 (0.46)	-14.87(28)	-3.71 (-4.22, -3.20)	0.000
	WPC–L	37.17 (0.85)			
Lysine	WPC	102.11 (1.45)	-7.55 (28)	-4.32 (-5.49, -3.15)	0.000
	WPC–L	106.43 (1.68)			
Histidine	WPC	18.97 (0.35)	-11.24 (28)	-1.62 (-1.92, -1.32)	0.000
	WPC–L	20.59 (0.43)			

Arginine	WPC	25.85 (0.70)	-10.08 (28)	-2.81 (-3.38, -2.24)	0.000
	WPC–L	28.66 (0.82)			
Cystine	WPC	28.29 (0.55)	-0.32 (28)	-0.07 (-0.53, 0.39)	0.753
	WPC–L	28.36 (0.67)			
Methionine	WPC	25.00 (0.57)	9.64 (28)	1.85 (1.46, 2.24)	0.000
	WPC–L	23.15 (0.48)			
Tryptophan	WPC	23.82 (0.85)	-17.44 (28)	-4.74 (-5.30, -4.18)	0.000
	WPC–L	28.56 (0.62)			

1. Mean (standard deviation)
2. t statistics (degrees of freedom)
3. Mean difference (95% confidence interval)
4. Independent t test

Table S3. t-Test for whey protein isolate (WPI) versus whey protein concentrate high fat (WPC–HF) (mg/g protein)

Amino acid	Product	M (sd)[1]	t (df)[2]	MD (95% C.I[3])	P value[4]
Aspartic Acid	WPI	124.70 (2.89)	8.60 (28)	9.33 (7.11, 11.55)	0.000
	WPC-HF	115.37 (3.04)			
Threonine	WPI	54.67 (1.52)	-33.13 (28)	-24.57 (-26.08, -23.05)	0.000
	WPC-HF	79.24 (2.43)			
Serine	WPI	38.27 (1.51)	-42.14 (28)	-19.95 (-20.92, -18.98)	0.000
	WPC-HF	58.22 (1.04)			
Glutamic Acid	WPI	188.07 (6.07)	11.29 (20.71)	19.82 (16.17, 23.48)	0.000
	WPC-HF	168.24 (3.07)			
Proline	WPI	45.50 (1.84)	-13.80 (28)	-14.88 (-17.08, -12.67)	0.000
	WPC-HF	60.38 (3.74)			
Glycine	WPI	16.98 (0.56)	-25.49 (28)	-4.82 (-5.21, -4.431)	0.000
	WPC-HF	21.80 (0.47)			
Alanine	WPI	58.26 (1.72)	13.12 (28)	7.20 (6.08, 8.32)	0.000
	WPC-HF	51.07 (1.25)			
Valine	WPI	55.54 (1.76)	-14.15 (20.26)	-7.14 (-8.20, -6.09)	0.000
	WPC-HF	62.68 (0.85)			
Isoleucine	WPI	62.08 (2.19)	0.92 (23.89)	0.62 (-0.77, 2.01)	0.369
	WPC-HF	61.47 (1.41)			
Leucine	WPI	141.52 (3.09)	35.96 (28)	39.21 (36.97, 41.44)	0.000
	WPC-HF	102.31 (2.88)			
Tyrosine	WPI	37.12 (0.65)	20.20 (28)	5.53 (4.97, 6.09)	0.000
	WPC-HF	31.60 (0.84)			
Phenylalanine	WPI	37.04 (0.66)	11.39 (28)	2.36 (1.93, 2.78)	0.000
	WPC-HF	34.69 (0.46)			
Lysine	WPI	116.36 (2.20)	30.90 (28)	24.22 (22.62, 25.83)	0.000
	WPC-HF	92.14 (2.09)			
Histidine	WPI	19.34 (0.69)	-1.66 (21.15)	-0.33 (-0.75, 0.09)	0.113
	WPC-HF	19.67 (0.36)			

Arginine	WPI	24.48 (1.03)	-8.75 (28)	-2.92 (-3.61, -2.24)	0.000
	WPC-HF	27.41 (0.79)			
Cystine	WPI	35.50 (1.38)	24.88 (28)	10.91 (10.01, 11.81)	0.000
	WPC-HF	24.59 (0.99)			
Methionine	WPI	25.76 (1.13)	25.28 (28)	8.97 (8.24, 9.70)	0.000
	WPC-HF	16.79 (0.79)			
Tryptophan	WPI	30.19 (1.93)	9.59 (22.71)	5.55 (4.35, 6.75)	0.000
	WPC-HF	24.63 (1.14)			

1. Mean (standard deviation)
2. t statistics (degrees of freedom)
3. Mean difference (95% confidence interval)
4. Independent t test

Table S4. t-Test for demineralized whey protein (D90) versus cheese whey protein concentrate (WPC–C) (mg/g protein)

Amino acid	Product	M (sd)[1]	T[2] (df)[3]	MD (95% C.I[3])	P value[4]
Aspartic Acid	D90	107.88 (7.75)	6.21 (15.28)	12.71 (17.07, 8.36)	0.000
	WPC-C	120.59 (1.66)			
Threonine	D90	69.37 (2.87)	16.01 (20.90)	-13.32 (15.05, 11.59)	0.000
	WPC-C	82.68 (1.47)			
Serine	D90	49.13 (2.70)	11.26 (21.48)	-8.92 (10.57, 7.27)	0.000
	WPC-C	58.06 (1.45)			
Glutamic Acid	D90	172.64 (11.59)	5.41 (15.18)	-16.53 (23.03, 10.03)	0.000
	WPC-C	189.17 (2.38)			
Proline	D90	63.88 (8.30)	-0.75 (14.90)	-1.63 (-6.28, 3.02)	0.467
	WPC-C	65.51 (1.49)			
Glycine	D90	18.11 (0.68)	-12.82 (28)	-2.47 (-2.87, -2.08)	0.000
	WPC-C	20.59 (0.31)			
Alanine	D90	50.55 (2.31)	-13.13 (28.00)	-8.39 (-9.70, -7.08)	0.000
	WPC-C	58.95 (0.90)			
Valine	D90	57.32 (1.84)	-8.00 (28.00)	-6.07 (-7.62, -4.51)	0.000
	WPC-C	63.39 (2.29)			
Isoleucine	D90	58.67 (2.11)	-8.19 (28.00)	-7.29 (-9.12, -5.46)	0.000
	WPC-C	65.97 (2.73)			
Leucine	D90	103.85 (3.50)	-10.38 (19.01)	-10.20 (-12.26, -8.15)	0.000
	WPC-C	114.05 (1.50)			
Tyrosine	D90	27.72 (6.51)	-2.01 (14.21)	-3.39 (-7.00, 0.22)	0.064
	WPC-C	31.11 (0.56)			
Phenylalanine	D90	31.51 (1.12)	-6.23 (18.59)	-1.94 (-2.60, -1.29)	0.000
	WPC-C	33.46 (0.46)			
Lysine	D90	88.19 (5.95)	-8.80 (15.65)	-13.92 (-17.28, -10.56)	0.000
	WPC-C	102.11 (1.45)			
Histidine	D90	16.49 (2.75)	-3.46 (14.46)	-2.48 (-4.01, -0.95)	0.004
	WPC-C	18.97 (0.35)			
Arginine	D90	24.67 (2.51)	1.77 (16.18)	-1.19 (-2.61, 0.24)	0.096

	WPC-C	25.85 (0.70)			
Cystine	D90	25.33 (3.57)	3.18 (14.67)	-2.96 (-4.95, -0.97)	0.006
	WPC-C	28.29 (0.55)			
Methionine	D90	20.40 (0.76)	-18.66 (28.00)	-4.60 (-5.10, -4.09)	0.000
	WPC-C	25.00 (0.57)			
Tryptophan	D90	19.17 (1.78)	-9.11 (28)	-4.65 (-5.70, -3.60)	0.000
	WPC-C	23.82 (0.85)			

1 Mean (standard deviation)
2 t statistics,
3 degrees of freedom,
4 95% confidence interval
5 Independent t test

Table S5. t-Test for demineralized whey protein (D90) versus cheese whey protein concentrate (WPC–C) (AA %Total).

Amino acid	Product	M (sd)[1]	T[2] (df)[3]	MD (95% C.I[3])	P value[4]
Aspartic Acid	D90	10.73 (0.44)	0.29 (14.82)	0.03 (-0.21, 0.28)	0.776
	WPC-C	10.70 (0.08)			
Threonine	D90	6.91 (0.37)	-4.32 (15.65)	-0.42 (-0.63, -0.21)	0.001
	WPC-C	7.33 (0.09)			
Serine	D90	4.89 (0.19)	-4.42 (28.00)	-0.26 (-0.38, -0.14)	0.000
	WPC-C	5.15 (0.13)			
Glutamic Acid	D90	17.17 (0.60)	2.31 (18.82)	0.39 (0.04, 0.74)	0.032
	WPC-C	16.78 (0.25)			
Proline	D90	6.34 (0.61)	3.29 (15.11)	0.53 (0.19, 0.88)	0.005
	WPC-C	5.81 (0.12)			
Glycine	D90	1.80 (0.07)	-1.16 (16.18)	-0.02 (-0.06, 0.02)	0.263
	WPC-C	1.83 (0.02)			
Alanine	D90	5.03 (0.22)	-3.33 (15.56)	-0.20 (-0.32, -0.07)	0.004
	WPC-C	5.23 (0.05)			
Valine	D90	5.71 (0.16)	1.59 (28.00)	0.09 (-0.03, 0.20)	0.123
	WPC-C	5.62 (0.15)			
Isoleucine	D90	5.84 (0.20)	-0.10 (28.00)	-0.01 (-0.15, 0.14)	0.925
	WPC-C	5.84 (0.19)			
Leucine	D90	10.34 (0.18)	4.80 (15.64)	0.22 (0.13, 0.32)	0.000
	WPC-C	10.11 (0.04)			
Tyrosine	D90	2.74 (0.55)	-0.12 (14.07)	-0.02 (-0.32, 0.29)	0.908
	WPC-C	2.76 (0.03)			
Phenylalanine	D90	3.14 (0.08)	7.74 (16.50)	0.17 (0.13, 0.22)	0.000
	WPC-C	2.97 (0.03)			
Lysine	D90	8.79 (0.72)	-1.40 (14.12)	-0.26 (-0.66, 0.14)	0.185
	WPC-C	9.06 (0.05)			
Histidine	D90	1.65 (0.31)	-0.41 (14.08)	-0.03 (-0.20, 0.14)	0.686
	WPC-C	1.68 (0.02)			
Arginine	D90	2.45 (0.17)	3.57 (15.82)	0.16 (0.06, 0.25)	0.003

	WPC-C	2.29 (0.04)			
Cystine	D90	2.52 (0.27)	0.09 (15.32)	0.01 (-0.15, 0.16)	0.928
	WPC-C	2.51 (0.06)			
Methionine	D90	2.04 (0.14)	-4.65 (18.52)	-0.18 (-0.26, -0.10)	0.000
	WPC-C	2.22 (0.06)			
Tryptophan	D90	1.91 (0.22)	-3.33 (16.63)	-0.20 (-0.32, -0.072)	0.004
	WPC-C	2.11 (0.07)			

1. Mean (standard deviation)
2. t statistics,
3. degrees of freedom,
4. 95% confidence interval
5. Independent t test
